# Epidemiological characteristics and spatiotemporal patterns of scrub typhus in Yunnan Province from 2006 to 2017

**DOI:** 10.1038/s41598-022-07082-x

**Published:** 2022-02-22

**Authors:** Pei-Ying Peng, Lei Xu, Gu-Xian Wang, Wen-Yuan He, Ting-Liang Yan, Xian-Guo Guo

**Affiliations:** 1 Qujing Medical College, Qujing, 655011 Yunnan Province People’s Republic of China; 2 Vector Laboratory, Institute of Pathogens and Vectors, Yunnan Provincial Key Laboratory for Zoonosis Control and Prevention, Dali University, Dali, Yunnan Province People’s Republic of China

**Keywords:** Infectious diseases, Epidemiology

## Abstract

Scrub typhus is an acute infectious disease in humans. A temporal, spatial and epidemiologic study was conducted to understand the characteristics of scrub typhus in Yunnan, to assist public health prevention and control measures. Based on the data on all cases reported in Yunnan during 2006–2017, we characterized the epidemiological features. Spatio-temporal patterns and Q-type cluster method were adopted to analyze the incidence of scrub typhus in Yunnan. In total, 27,838 scrub typhus cases were reported in Yunnan during 2006–2017. Of these, 49.53% (13,787) were male and 50.47% (14,051) were female (*P* > 0.05). Most patients were farmers (71.70%) (*P* < 0.05) and children aged 0–5 years (13.16%) (*P* < 0.01), which accounted for 84.86% of the total cases. An almost 20-fold increase in the number of patients was observed in 2017 (6,337 cases) compared to 2006 (307 cases). Baoshan and Lincang had the most cases accounting for 41.94%, while Diqing had the lowest incidence (only 3 cases). Sixteen municipalities infected were classified into three groups numbered in sequence. The incidence of scrub typhus in Yunnan is high and the annual incidence increased noticeably over time. Our results also indicate that surveillance and public education need to be focused on Baoshan, Lincang and Dehong.

## Introduction

Scrub typhus (tsutsugamushi disease) is an ancient infectious disease primarily endemic in several Asia–Pacific nations^1,2^. It is an infection caused by the pathogen *Orientia tsutsugamushi*, which can be transmitted occasionally to humans after being bitten by infected chigger mites (trombiculid mites)^3,4^. *O. tsutsugamushi* spreads throughout the body via the blood and lymphatic vessels, so that patients infected with this microorganism manifest a variety of clinical symptoms and signs such as myalgia and diffuse lymphadenopathy^5^. The larval stage of the mites (also known as chiggers) is the only parasitic stage that transmits the pathogen to people and other vertebrates^6,7^. Rodents are important to the maintenance of the disease in that they are known as incidental hosts for chigger mites^8^. Research shows that more than every fifth people are antibodies carriers in regions affected by scrub typhus, which is a sign of previous contact^9^. Up to now, however, there is still no point-of-care diagnostics available and no reliable and effective human vaccine against tsutsugamushi disease, which will pose a significant threat to public health^2^.

Before 1986, scrub typhus epidemic areas were only distributed in the south of Yangtze River, including provinces of Guangdong, Yunnan, Fujian and Zhejiang^10,11^. However, recent studies showed that the geographic distribution of the disease has expanded to northern China^12^. To date, according to the latest monitoring data, scrub typhus has spread to all over China except Ningxia and Shanghai and the incidence has increased rapidly in recent years^13^. The number of reported cases of scrub typhus increased rapidly from 2006 to 2016, increasing by 15.4 times within 11 years. China reported 22,558 cases (including suspected cases), with a reported incidence rate was 1.64/100,000 people in 2016^13^. The scrub typhus epidemics in Guangdong and Yunnan are the most serious^11^.

Scrub typhus cases in Yunnan province were confirmed by Dr Wei Xi using serological methods in the year of 1943^14^. The disease is widely distributed in 16 municipalities or prefectures of Yunnan province^15,16^. In recent years, local outbreaks of scrub typhus have occurred in many areas of Yunnan, and recently identified endemic foci of scrub typhus are reported to be expanding^17–19^. The increased incidence of tsutsugamushi disease in Yunnan offers an opportunity to enhance our understanding of the epidemiological trends of this re-emerging infectious disease. The results from our study will provide authentic and reliable evidence for local health-care authorities to design strategies to alleviate the health effects of scrub typhus.

## Results

### Descriptive analysis of scrub typhus in Yunnan province

**Figure 1:**
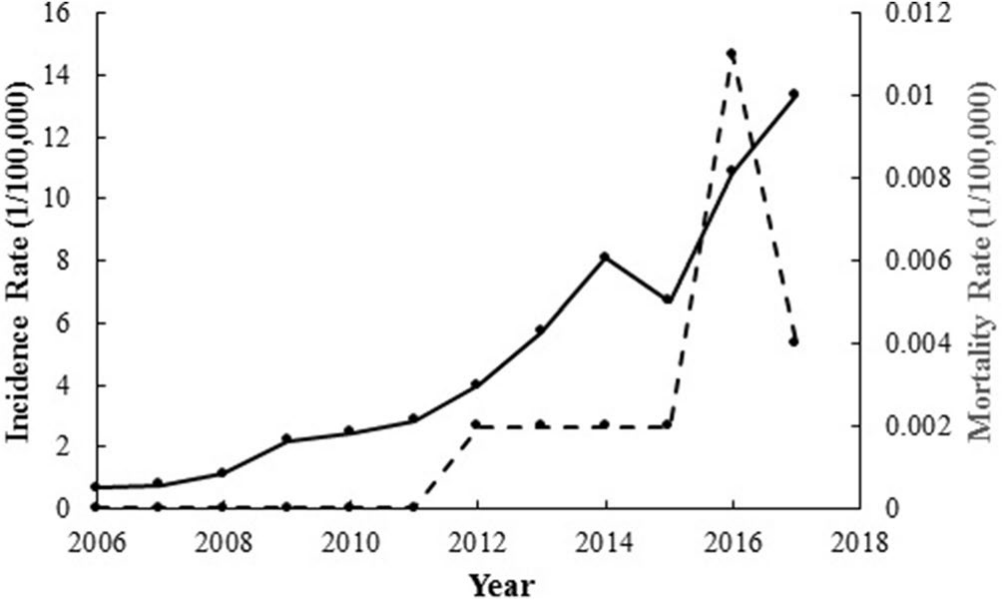
Fluctuation of epidemic features of scrub typhus cases throughout the year in Yunnan province, China. The straight line represents the incidence rate (cases per 100,000 population); and the broken line represents the mortality rate (cases per 100,000 population).

A total of 27,838 clinically diagnosed and laboratory confirmed cases of scrub typhus were reported in Yunnan province during 2006–2017, among whom 11 patients died. The overall case fatality was 0.04%. The annual incidence ranged from 0.65/100,000 to 13.36/100,000, with a rapid uptrend observed year by year and with an average annual incidence rate of 4.87/100,000. Natural focus expansion was found. The number of confirmed scrub typhus cases peaked in 2017, when there were 20.64 times more reported cases than in 2006. Scrub typhus cases, the incidence rate (cases per 100,000 population) and the mortality rate (cases per 100,000 population) have been increasing rapidly from 2006 to 2017 in Yunnan province, southwest China (Table 1, Fig. 1).

**Table 1:** Epidemiologic features of scrub typhus cases in Yunnan province China, 2006–2017.

Year	No. of cases (%)	Incidence rate* (cases per 100,000 population)	No. of deaths (%)	Mortality rate (cases per 100,000 population)	CFR^#^ (%)
2006	307 (1.10)	0.65	0 (0)	0	0
2007	365 (1.31)	0.77	0 (0)	0	0
2008	526 (1.89)	1.11	0 (0)	0	0
2009	1,045 (3.75)	2.20	0 (0)	0	0
2010	1,155 (4.15)	2.44	0 (0)	0	0
2011	1,346 (4.84)	2.84	0 (0)	0	0
2012	1,884 (6.77)	3.97	1 (9.09)	0.002	0.05
2013	2,707 (9.72)	5.71	1 (9.09)	0.002	0.04
2014	3,836 (13.78)	8.09	1 (9.09)	0.002	0.03
2015	3,176 (11.41)	6.70	1 (9.09)	0.002	0.03
2016	5,154 (18.51)	10.87	5 (45.45)	0.011	0.10
2017	6,337 (22.76)	13.36	2 (18.18)	0.004	0.03
Total	27,838 (100.00)	4.87	11 (100.00)	0.002	0.04

*Annual average incidence.

^#^Case fatality rate.

The scrub typhus cases occurred in all 16 municipalities or prefectures of Yunnan, and the cases were reported from 118 of 129 counties. Baoshan municipality and Lincang municipality had the most cases accounting for 41.94%, while only 3 cases were confirmed in Diqing prefecture. Farmers were the primarily affected occupation, accounting for 68.41% (19,045 cases), followed by students aged 0–6 years (2650 cases, 9.52%) and young children under the age of five (4867 cases, 17.48%). The total number of cases of these three groups of scrub typhus was 26,562, accounting for 95.42% of the cases. While sex-specific scrub typhus incidence from 2006 to 2017 did not change significantly over time, the ratio of male (49.53%) to female (50.47%) was 0.98:1 (*P* > 0.05).

### Time-series analyses for scrub typhus

**Figure 2:**
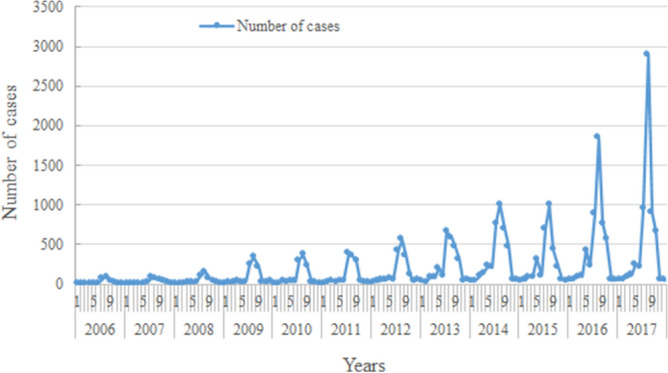
Seasonal distribution fluctuation of scrub typhus cases reported of Yunnan province from 2006 to 2017.

**Figure 3:**
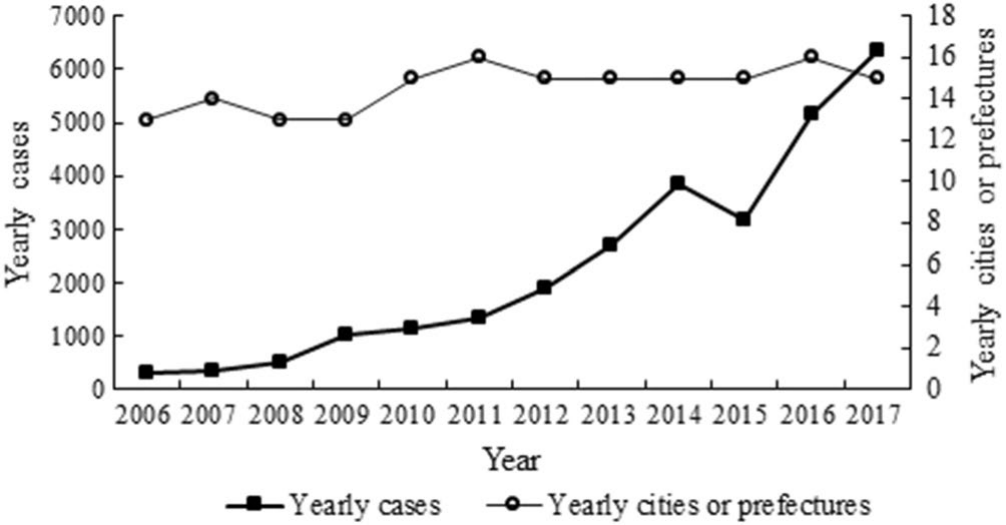
Time-series mapping of yearly scrub typhus.

Scrub typhus showed a seasonal pattern. The peak of monthly scrub typhus cases was in August every year. And the majority of confirmed cases of scrub typhus during 2006–2017 in Yunnan occurred yearly were mainly from July to October, which accounting for 79.22% (22,053/27,838) (Fig. 2). There were 27,838 cases recorded during 2006–2017. Annual cases and the annual number of municipalities or prefectures with cases increased year by year (Fig. 3). The yearly cases increased from 307 to 6337 in 2006–2017. The number of municipalities (or prefectures) with cases increased from 13 to 16 during 2006–2017, which also means that Yunnan is a province with a high incidence of scrub typhus.

### Spatial distribution analyses for scrub typhus

**Figure 4:**
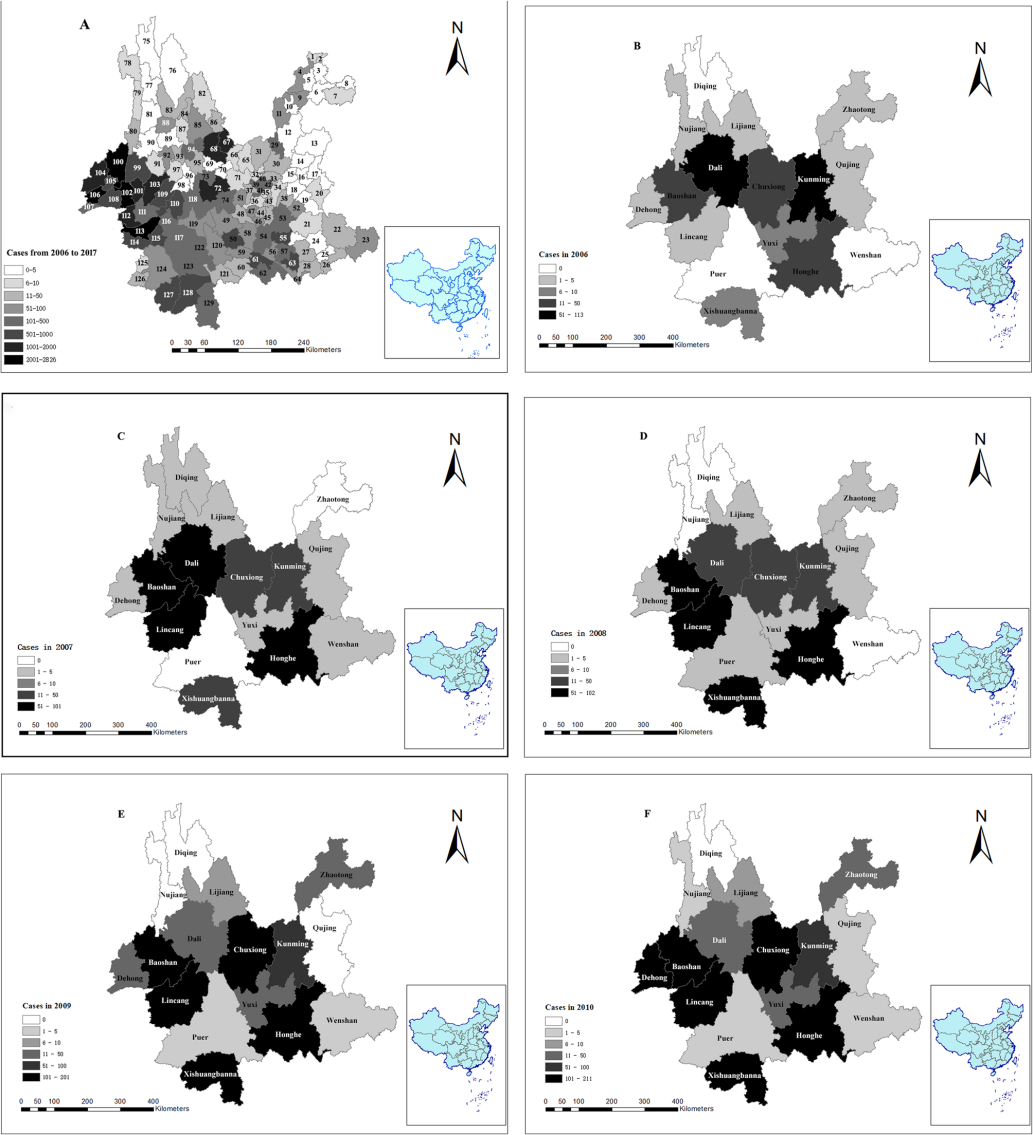

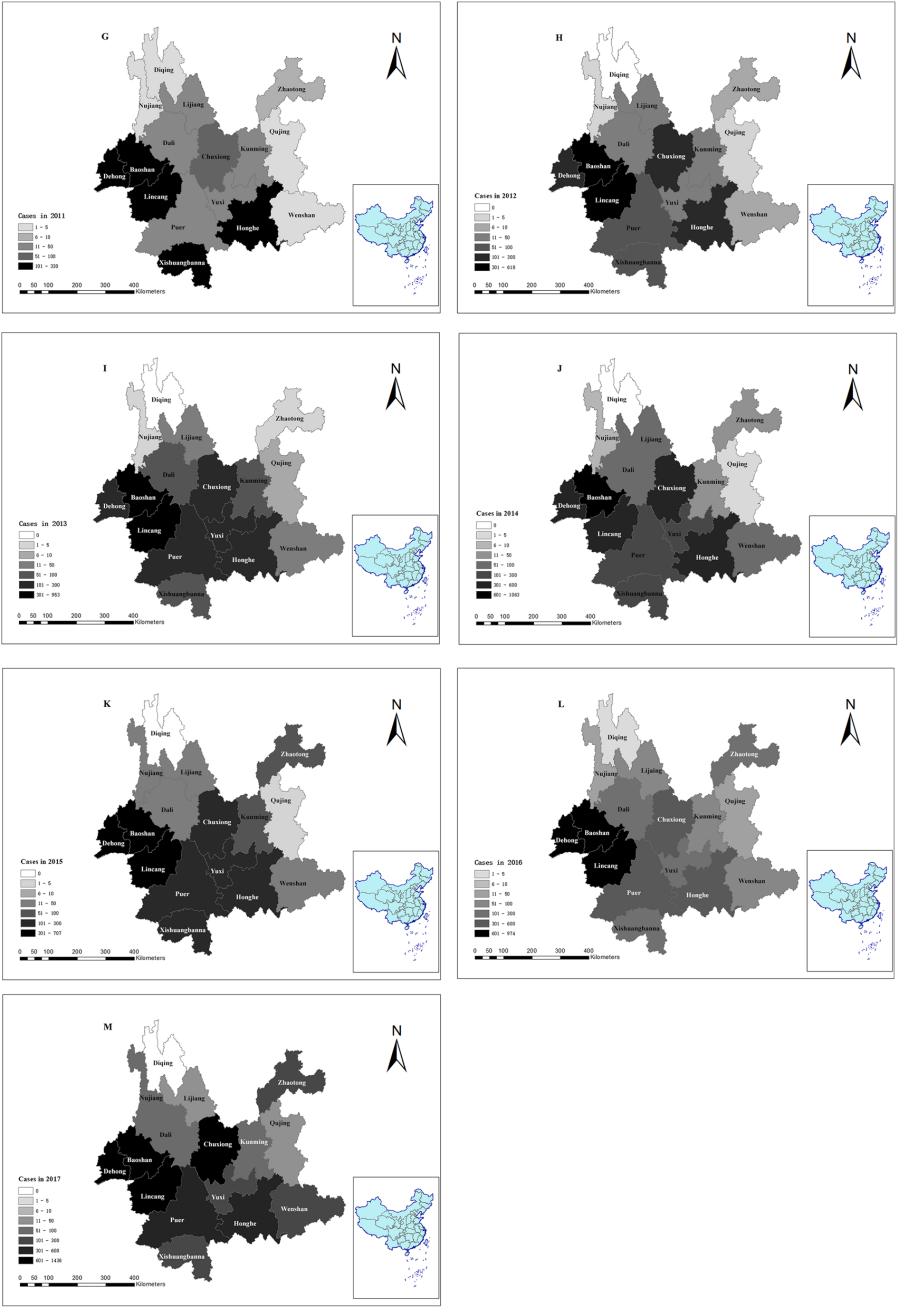
Spatial distribution analyses for scrub typhus in 2006–2017. (**A**) in twelve years in county level; (**B**) in 2006; (**C**) in 2007; (**D**) in 2008; (**E**) in 2009; (**F**) in 2010; (**G**) in 2011; (**H**) in 2012; (**I**) in 2013; (**J**) in 2014; (**K**) in 2015; (**L**) in 2016; (**M**) in 2017. Figure 4 was created for this manuscript using ArcGIS software (ArcGIS 10.6). Data for scrub typhus cases from 2006 to 2017 were provided by the Yunnan Provincial Notifiable Disease Report System. The limits and borders of Yunnan province and China were constructed using the geographical information from the National Geomatics Center of China (NGCC), and the website of NGCC is http://ngcc.cn/ngcc/. The (**A**) comprised of 129 counties (municipalities/ districts). Each number corresponds to a county (municipality/ district). We use the capital letter combination of county (municipality/ district) names to represent. **1–11** (Zhaotong): 1 (SJ), 2 (SF), 3 (YJ), 4 (YS), 5 (DG), 6 (YL), 7 (ZX), 8 (WX), 9 (ZY), 10 (LD), 11 (QJ). **12–20** (Qujing): 12 (HZ), 13 (XW), 14 (ZY), 15 (ML), 16 (QL), 17(FY), 18 (LL), 19(SZ), 20 (LP). **21–28** (Wenshan): 21 (QB), 22 (GN), 23 (FN), 24 (YS), 25 (XC), 26 (MLP), 27 (WS), 28 (MG). **29–42** (Kunming): 29 (DC), 30 (XD), 31(LQ), 32 (FM), 33 (SM), 34 (YL), 35 (CG), 36 (JN), 37 (AN), 38 (SL), 39 (WH), 40 (PL), 41 (XS), 42 (GD). **43–51** (Yuxi): 43 (CJ), 44 (JC), 45 (HN), 46 (TH), 47 (HT), 48 (ES), 49 (XP), 50 (YJ) 51(YM). **52–64** (Honghe): 52 (LX), 53 (ML), 54 (JS), 55 (KY), 56 (GJ), 57 (MZ), 58 (SP), 59 (HH), 60 (LC), 61 (YY), 62 (JP), 63 (PB), 64 (HK). **65–74** (Chuxiong): 65 (WD), 66 (YM), 67 (YR), 68 (DY), 69 (YA), 70 (MD), 71 (LF), 72 (CX), 73 (NH), 74 (SB). **75–77** (Diqing): 75 (DQ), 76 (XGLL), 77 (WX). **78–81** (Nujiang): 78 (GS), 79 (FG), 80 (LS), 81 (LP). **82–86** (Lijiang): 82 (NL), 83 (YL), 84 (GC), 85 (YS), 86 (HP). **87–98** (Dali): 87 (HQ), 88 (JC), 89 (EY), 90 (YL), 91(YP), 92 (YB), 93 (DL), 94 (BC), 95 (XY), 96 (MD), 97 (WS), 98 (NJ). **99–103** (Baoshan): 99 (LY), 100 (TC), 101 (SD), 102 (LL), 103 (CN). **104–108** (Dehong): 104 (YJ), 105 (LH), 106 (LC), 107 (RL), 108 (M). **109–116** (Lincang): 109 (FQ), 110 (Y), 111(YD), 112 (ZK), 113 (GM), 114 (CY), 115 (SJ), 116 (LX). **117–126** (Puer): 117 (JG), 118 (JD), 119 (ZY), 120 (MJ), 121 (JC), 122 (NE), 123 (SM), 124 (LC), 125 (XM), 126 (ML). **127**–**129** (Xishuangbanna): 127 (MH), 128 (JH), 129 (ML).

Scrub typhus occurred in 118 counties and 16 municipalities (or prefectures) from 2006 to 2017. There were more than 2000 cases in Longling County in Baoshan municipality, which was the county with the largest number of scrub typhus cases in Yunnan province. Number of scrub typhus cases ranged from 0 to 2826 in the county level in Yunnan province during 2006–2017 (Fig. 4A). The results of spatial distribution for scrub typhus from 2006 to 2017 are shown in Fig. 4(B–M).

**Figure 5:**
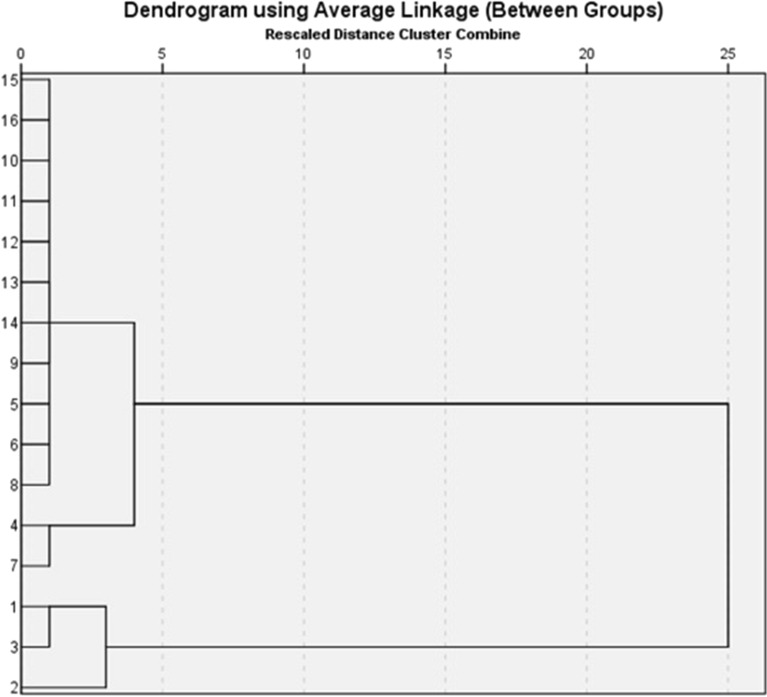
Hierarchical clustering dendrogram on the incidence of scrub typhus in Yunnan province, China (2006–2017).

The goal of hierarchical cluster analysis is to build a tree diagram where the cards that were viewed as most similar by the participants in the study are placed on branches that are close together. For example, in our study, Fig. 5 shows the result of a hierarchical cluster analysis of the data in Table 2. The key to interpreting a hierarchical cluster analysis is to look at the point at which any given pair of cards “join together” in the tree diagram. Cards that join together sooner are more similar to each other than those that join together later. The results of hierarchical cluster analysis on the incidence of scrub typhus are described in Fig. 5. Sixteen municipalities or prefectures of Yunnan province are classified into three groups numbered in sequence. Baoshan municipality (1), Lincang municipality (2) and Dehong Prefecture (3), which had the highest incidence of scrub typhus formed the first group in the clustering dendrogram. Chuxiong Prefecture (4), Honghe Prefecture (5), Puer municipality (6), Xishuangbanna Prefecture (7), Yuxi municipality (8), Dali Prefecture (9) and Nujiang Prefecture (14) formed the second separated clustering group. The third group with the lowest incidence of scrub typhus, is Kunming municipality (10), Zhaotong municipality (11), Wenshan Prefecture (12), Lijiang municipality (13), Qujing municipality (15) and Diqing Prefecture (16) (Table 2, Fig. 5).

**Table 2:** Incidence of scrub typhus in Yunnan Province, China (2006–2017, cases per 100,000 population).

Area	2006	2007	2008	2009	2010	2011	2012	2013	2014	2015	2016	2017
1	0.56	2.11	4.07	7.18	8.42	13.17	24.66	38.03	42.42	28.21	38.87	53.03
2	0.21	2.35	3.37	6.71	7.24	10.49	16.50	19.84	22.30	24.28	39.18	59.09
3	0.17	0.08	0.17	4.05	9.66	18.41	16.02	21.14	38.98	45.75	67.13	74.57
4	1.30	0.63	1.79	4.99	5.40	3.09	4.47	5.77	19.63	9.87	20.86	25.67
5	0.80	2.24	2.16	3.38	4.38	3.35	3.58	5.15	7.07	4.20	7.40	9.78
6	0.00	0.00	0.04	0.08	0.08	0.55	3.34	4.17	5.11	8.65	15.97	18.80
7	0.53	1.76	8.02	17.72	9.35	11.29	5.11	6.35	10.05	10.76	14.46	11.11
8	0.26	0.13	0.17	0.61	1.52	1.30	1.87	5.34	8.94	6.34	11.28	8.38
9	3.27	1.74	0.87	1.07	0.72	0.84	0.93	1.65	2.29	0.75	2.98	2.20
10	1.06	0.45	0.58	0.79	0.90	0.44	0.54	0.81	0.75	0.87	1.40	1.21
11	0.02	0.00	0.02	0.33	0.77	0.12	0.17	0.06	0.61	1.50	2.34	3.30
12	0.00	0.03	0.00	0.11	0.14	0.06	0.28	0.80	2.42	1.34	2.81	2.93
13	0.08	0.08	0.40	0.48	0.72	1.12	2.73	3.45	5.94	3.05	4.34	3.94
14	0.19	0.19	0.00	0.00	0.37	0.37	0.75	0.75	1.12	3.37	7.68	9.93
15	0.03	0.03	0.02	0.00	0.02	0.05	0.03	0.15	0.07	0.09	0.20	0.38
16	0.00	0.25	0.00	0.00	0.00	0.25	0.00	0.00	0.00	0.00	0.25	0.00

1. Baoshan municipality; 2. Lincang municipality; 3. Dehong Prefecture; 4. Chuxiong Prefecture; 5. Honghe Prefecture; 6. Puer municipality; 7. Xishuangbanna Prefecture; 8.Yuxi municipality; 9. Dali Prefecture; 10. Kunming municipality; 11. Zhaotong municipality; 12. Wenshan Prefecture; 13. Lijiang municipality; 14. Nujiang Prefecture; 15. Qujing municipality; 16. Diqing Prefecture.

## Discussion

In China, scrub typhus was removed from the National Notifiable Disease Reported System since 1989. It was legally redefined as a notifiable disease in 2006 and must be reported to the China Center for Disease Control and Prevention. The number of the cases of scrub typhus reported has been on the rise since 2006^20^. In our study, the results indicate that from 2006 to 2017 there has been an alarming increase in the number of reported cases of scrub typhus in Yunnan province, indicating that scrub typhus remains an important public health problem throughout the province. Given the trends identified in this study, our findings suggest that scrub typhus incidence is likely to continue to increase in the future.

Research shows that chigger mites are the only vector of tsutsugamushi disease^21,22^. The complicated topographic landform and high biodiversity of Yunnan may contribute the extremely high species diversity of chigger mites in this province^23^, which indirectly leads to a large number of scrub typhus cases. There are several possible factors to explain the rapid increase of cases in Yunnan: (1) Located in the southwest of China, Yunnan is a mountainous province with a vast territory, complicated topographic landform, and high biodiversity^24,25^. The geographical location with unique landscape, complex topography, and diversified ecological environment of Yunnan provide the unique natural landscape resources that are attractive for tourism, especially eco-tourism. With the development of Yunnan tourism, tourism has expanded into forests and grasslands, which means more people have a chance of getting infected by chiggers; (2) The number of farmers working in the field, field operators (logging, road construction workers, geological surveyors, etc.), field training units and field tourists have increased. Therefore, they are more likely to be attacked by chigger mites and are prone to infection. (3) People living in Yunnan, a province where education is undeveloped, have poor knowledge of scrub typhus. (4) There are abundant species of plants and animals with a plenty of biologically diverse gene resources in Yunnan, which has been one of the hot places of biodiversity conservation in China^26,27^. In addition, in Yunnan there are three rivers (Jinshajiang River, Lancangjiang River, and Nujiang River) flowing from the northwest towards the southwest. The three rivers parallel to each other, forming the “Protected Area of Three Parallel Rivers”, which is a famous “World Natural Heritage Area”. It is considered as a hotspot of biodiversity with high species diversity in Asian continent^28,29^. The geographical location with unique landscape, complex topography, and diversified ecological environment of Yunnan provide a good place to the survival of chigger mites.

However, in our study, it has been observed there is a very low incidence of scrub typhus in Diqing prefecture over the studied years. Relevant literature provided evidence that climatic factors could be important factors associated with occurrence of scrub typhus. Temperature and rainfall were positively associated with scrub typhus incidence^30,31^. Research shows that each one degrees centigrade rise in temperature corresponded to an increase of 14.98% in the monthly number of scrub typhus cases and a 1 mm rise in rainfall corresponded to an increase of 0.05% or 0.10% in the monthly number of scrub typhus cases^32^. Also, the study of chigger mites shows that warm and humid climate may be beneficial to the growth and reproduction of chigger mites^21^. And in our previous study, in total, 274 species and 120,138 individuals of chigger mites were collected in 29 investigation sites of Yunnan province in 2001–2013. However, only two species of vector chigger mites (*Leptotrombidium scutellare* and *L. insulare*) and 3846 individuals of chigger mites were collected in Diqing prefecture, which accounting for 3.20% (3846/120,138) of all the chigger mites. The species diversity of chigger mites was much lower in high-altitude regions (> 3500 m) than in middle-altitude regions^6,23^. Overall, the low temperature, little rainfall and very high altitude in Diqing prefecture are not suitable for the survival of chigger mites (or vector chigger mite species), which are the reasons why very low incidence of scrub typhus in Diqing over the studied years.

The results of our study identified seasonal differences in the onset and duration of tsutsugamushi disease that suggest the existence of both summer and autumn transmission of scrub typhus (summer-autumn type) in Yunnan, which is not in accordance with scrub typhus type in other provinces of China^33–35^. In our study farmers and children aged less than 5 years old were the groups most at risk due to lower awareness of scrub typhus, poor protection during agriculture or field recreational activities and reduced use of protective measures when playing in grassland or woods for children^36^.

The results of our study indicated that from 2006 to 2017 scrub typhus occurred mainly in Baoshan municipality, Lincang municipality, Dehong Prefecture, Chuxiong Prefecture and Honghe Prefecture. The classification by hierarchical cluster analysis has produced results that correspond to the rate of incidence of scrub typhus in Yunnan province. Our results also indicated that surveillance systems, public education and public health efforts to control and prevent scrub typhus need to be focused on the areas with highest incidence. Baoshan municipality, Lincang municipality and Dehong Prefecture were the areas with the highest incidence of scrub typhus in Yunnan province. Located in southwest of the province, the three municipalities have high temperature, intense sunshine, abundant rainfall and relatively low atmospheric pressure, which made an ideal environment for chigger mites^37,38^.

This study analyzed the epidemiologic characteristics and spatio-temporal pattern for scrub typhus in Yunnan during 2006–2017. In conclusion, the incidence of tsutsugamushi disease in Yunnan province is on the rise, and the epidemic area is expanding. We should strengthen the ability of early diagnosis and detection, carry out health education, monitor the epidemic situation and risk factors of tsutsugamushi disease, and prevent the harm of tsutsugamushi disease. A set of preventive strategies including public health education and personal protection facilities should be promoted in high-risk populations (the elderly, farmers and children aged less than 5 years old) and municipalities (Baoshan municipality, Lincang municipality and Dehong Prefecture) in Yunnan province. Attention should also be focused on the relationship between the potential factors that may influence scrub typhus, such as climatic, geographical and environmental factors, and explore the underlying reasons for the rise and spread of scrub typhus in Yunnan province.

## Methods

### Study area

Yunnan province, an inland province at a low latitude and high elevation, lying between 21°8′ ~ 29°15′N and 97°31′ ~ 106°11′E in southwestern China, has a vast territory with diverse and unique natural resources. Located in the southwest of China, Yunnan harbors around 45.97 million residents in its 16 municipalities or prefectures with a total land area of nearly 394,000 km^2^ (http://stats.yn.gov.cn/tjsj/tjnj/201912/t20191202_908222.html). It mainly features a subtropical plateau monsoon climate type. Mean temperature for the hottest month in Yunnan (typically in July) is 19 °C–22 °C, while mean temperature for the coldest month (typically in January) is 6 °C–8 °C. The annual average precipitations in most areas is more than 1000 mm (http://www.yn.gov.cn/yngk/).

### Data collection and management

In China, scrub typhus is a notifiable disease that must be reported to the China Center for Disease Control and Prevention. The case definition for tsutsugamushi disease consists of an individual who has traveled to an endemic area or reported contact with chigger mites or rodents within three weeks before the onset of illness, along with clinical manifestations (such as high fever, lymphadenopathy, skin rash, and eschars or ulcers), and at least one of the following laboratory criteria: an agglutination titer ≥ 1:160 in the Weil–Felix test using the OX_K_ strain of *Proteus mirabilis*; a fourfold or more rise of antibody titer against *O. tsutsugamushi* using the indirect immunofluorescence antibody assay; detection of *O*. *tsutsugamushi* by PCR (polymerase chain reaction) in clinical specimens; or isolation of *O. tsutsugamushi* from clinical specimens^20^. Patients with other established causes of fever were excluded. A standard form was adopted by local physicians to collect individual information on each scrub typhus case, including epidemiological exposure histories, age, sex, address, date of onset, diagnosis, and laboratory test results. Routine case reporting is done by hospitals via the National Notifiable Disease Report System within 24 hours^20^. To describe epidemic characteristics and explore the incidence of clustering of confirmed cases of scrub typhus in Yunnan China at the municipality (prefecture) level, we used surveillance data reported confirmed scrub typhus cases from 2006 to 2017, routinely collected by the Yunnan Provincial Notifiable Disease Report System. Population data at the municipality (prefecture) level were obtained from the National Bureau of Statistics of China, which is based on the Sixth National Census in 2010.

### Statistical analysis

The differences in the incidence rates of scrub typhus cases by sex, age group, and occupation were analyzed using the chi-square test. *P* values < 0.05 were considered statistically significant. Q type cluster method of hierarchical cluster analysis method together with Z-score standardization and squared Euclidean distance were adopted in the calculation clustering analysis under the statistical software package SPSS (v.25). The clustering result was presented in a dendrogram. Time-series analyses for scrub typhus cases were conducted using IBM SPSS Statistics version 24.0 (IBM Corp., NY, USA). Spatial distribution analyses of scrub typhus every year were conducted using Spatial Mapping in ArcGIS version 10.6.

### Ethics approval and consent to participate

In research of present paper, the data of scrub typhus cases were extracted from online databases, and no sample of human and animal was included. Therefore, the ethical approval and consent to participate are not necessary for the research. Meanwhile, all data were anonymous, and we all confirmed that all methods were carried out in accordance with relevant guidelines and regulations.

## Data Availability

The datasets used and/or analyzed during the current study are available from the corresponding author on reasonable request.
